# Crystal structure of a one-dimensional coordination polymer of tin(IV) bromide with 1,4-di­thiane

**DOI:** 10.1107/S2056989015023932

**Published:** 2015-12-16

**Authors:** Hans Reuter, Natalia Röwekamp-Krugley, Marius Imwalle, Simona Keil, Martin Reichelt

**Affiliations:** a Institute of Chemistry of New Materials, University of Osnabrueck, Barbarstr. 7, 49069 Osnabrueck, Germany

**Keywords:** crystal structure, tin(IV) bromide, 1,4-di­thiane, coordination polymer, Br⋯Br inter­actions, Br⋯H inter­actions

## Abstract

The title compound, [SnBr_4_(C_4_H_8_S_2_)] {systematic name: *catena*-poly[[tetrabromidotin(IV)]-μ-1,4-dithiane-κ^2^ *S*:*S*′]}, represents the first 1,4-di­thiane complex with tin as coordination centre. The asymmetric unit consist of half a formula unit with the tin(IV) atom at the centre of symmetry at 0,0,1/2 (Wyckoff symbol *b*) and a centrosymmetric 1,4-di­thiane mol­ecule with the centre of symmetry in 1/2,0,1 (Wyckoff symbol *c*). The tin(IV) atom is coordinated in a distorted octa­hedral manner by the four bromine atoms and two sulfur atoms of two 1,4-di­thiane mol­ecules in a *trans*-position. Sn—Br [mean value: 2.561 (5) Å] and Sn—S distances [2.6546 (6) Å] are in the typical range for octa­hedrally coordinated tin(IV) atoms and the di­thiane mol­ecule adopts a chair conformation. The one-dimensional polymeric chains propagate along the [101] direction with weak inter­molecular Br⋯Br [3.5724 (4) Å] between parallel chains and weak Br⋯H inter­actions [2.944–2.993 Å] within the chains.

## Related literature   

For the structural parameters in macrocyclic thio­ether complexes with SnBr_4_, see: Levason *et al.* (2003 ▸), and for di­thio­ether complexes with SnBr_4_, see: Dann *et al.* (1996 ▸). For the oxidation of tin(II) to tin(IV), see: Deacon *et al.* (1997 ▸).
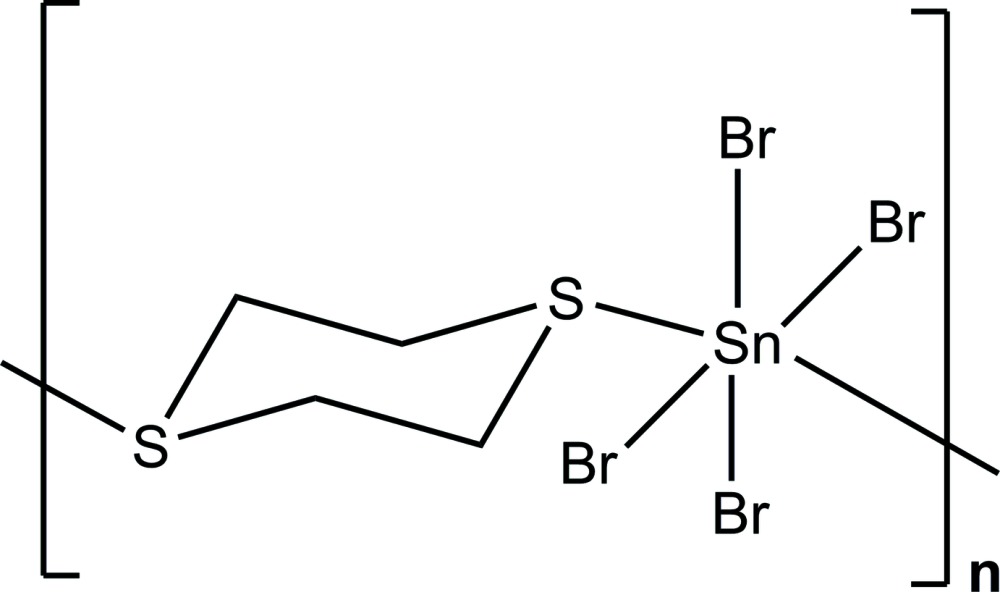


## Experimental   

### Crystal data  

[SnBr_4_(C_4_H_8_S_2_)]
*M* *_r_* = 558.55
Monoclinic, 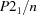

*a* = 7.1033 (4) Å
*b* = 12.0526 (8) Å
*c* = 7.4032 (5) Å
β = 112.144 (2)°
*V* = 587.06 (7) Å^3^
*Z* = 2
Mo *K*α radiation
μ = 16.09 mm^−1^
*T* = 100 K
0.16 × 0.06 × 0.06 mm

### Data collection  

Bruker APEXII CCD diffractometer
Absorption correction: multi-scan (*SADABS*; Bruker, 2009 ▸) *T*_min_ = 0.182, *T*_max_ = 0.450
22217 measured reflections
1426 independent reflections
1339 reflections with *I* > 2σ(*I*)
*R*_int_ = 0.066

### Refinement  

*R*[*F* ^2^ > 2σ(*F* ^2^)] = 0.017
*wR*(*F* ^2^) = 0.036
*S* = 1.14
1426 reflections
54 parameters
H-atom parameters constrained
Δρ_max_ = 0.72 e Å^−3^
Δρ_min_ = −0.46 e Å^−3^

### 

Data collection: *APEX2* (Bruker, 2009 ▸); cell refinement: *SAINT* (Bruker, 2009 ▸); data reduction: *SAINT*; program(s) used to solve structure: *SHELXS97* (Sheldrick, 2008 ▸); program(s) used to refine structure: *SHELXL2014*/7 (Sheldrick, 2015 ▸); molecular graphics: *DIAMOND* (Brandenburg, 2006 ▸) and *Mercury* (Macrae *et al.*, 2008 ▸); software used to prepare material for publication: *SHELXTL* (Sheldrick, 2008 ▸).

## Supplementary Material

Crystal structure: contains datablock(s) I, New_Global_Publ_Block. DOI: 10.1107/S2056989015023932/nr2064sup1.cif

**Fig. 1.**
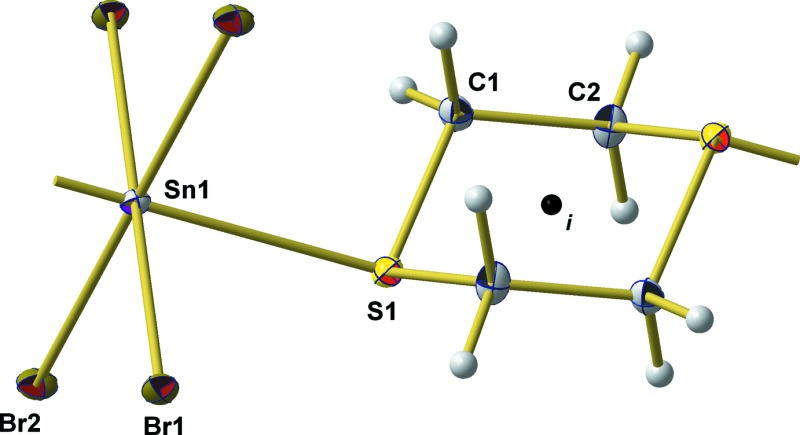
Ball-and-stick model of the asymmetric unit of the title compound with the atomic numbering scheme used. For a better understanding the asymmetric unit of the 1,4-dithiane molecule has been extended by its symmetry-related atoms generated by the centre of symmetry i (black dot) at 1/2,0,1. With exception of the H atoms, which are shown as spheres of arbitrary radius, all atoms are drawn as displacement ellipsoids at the 50% probability level.

Structure factors: contains datablock(s) I. DOI: 10.1107/S2056989015023932/nr2064Isup2.hkl

i . DOI: 10.1107/S2056989015023932/nr2064fig1.tif
Ball-and-stick model of the asymmetric unit of the title compound with the atomic numbering scheme used. For a better understanding the asymmetric unit of the 1,4-di­thiane mol­ecule has been extended by its symmetry-related atoms generated by the centre of symmetry *i* (black dot) at 1/2,0,1. With exception of the H atoms, which are shown as spheres of arbitrary radius, all atoms are drawn as displacement ellipsoids at the 50% probability level.

**Fig. 2.**
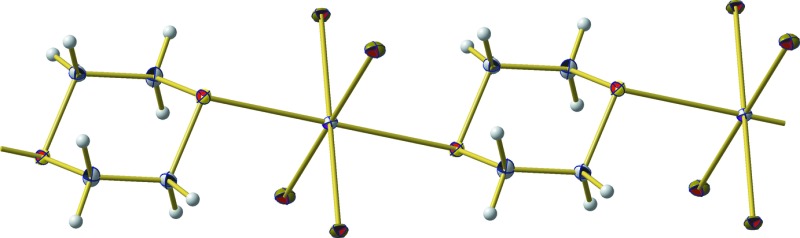
Part of the one-dimensional coordination polymer showing two complete building units.

**Fig. 3.**
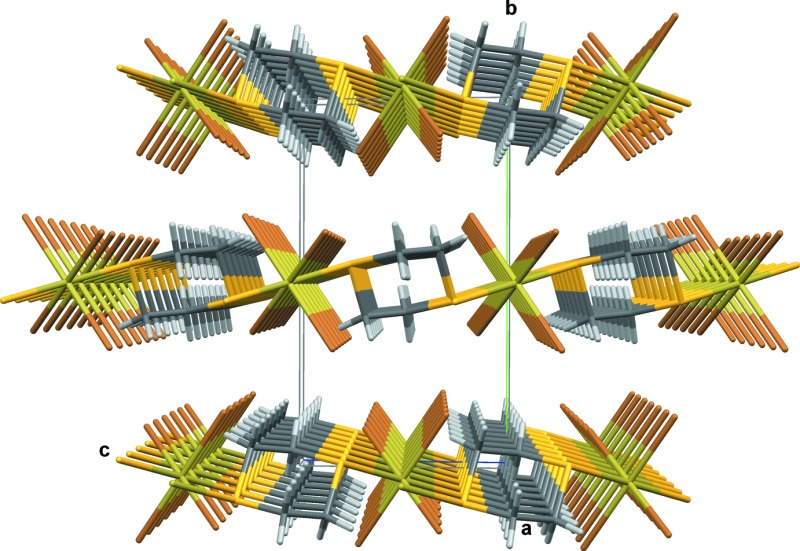
Perspective view of the crystal structure looking down the a axis.

. DOI: 10.1107/S2056989015023932/nr2064fig2.tif
Part of the one-dimensional coordination polymer showing two complete building units.

a . DOI: 10.1107/S2056989015023932/nr2064fig3.tif
Perspective view of the crystal structure looking down the *a* axis.

**Fig. 4.**
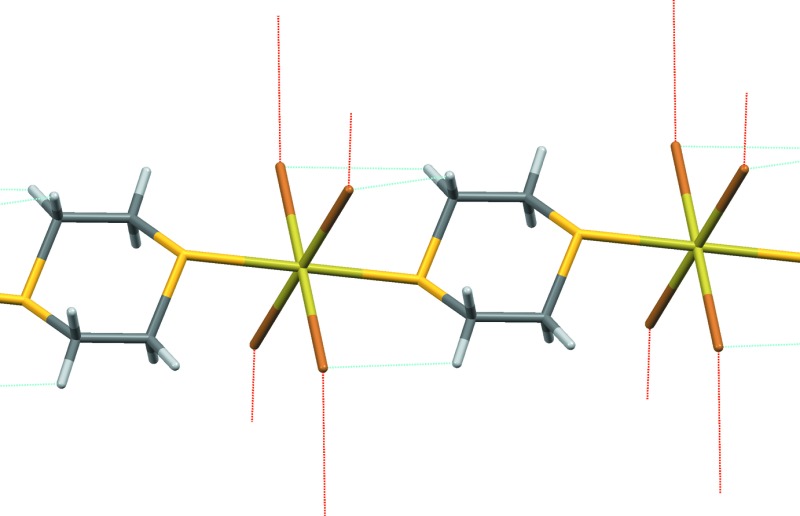
Shortest intrachain H···Br (blue) and interchain Br···Br (red) interactions.

. DOI: 10.1107/S2056989015023932/nr2064fig4.tif
Shortest intra­chain H⋯Br (blue) and inter­chain Br⋯Br (red) inter­actions.

**Fig. 5.**
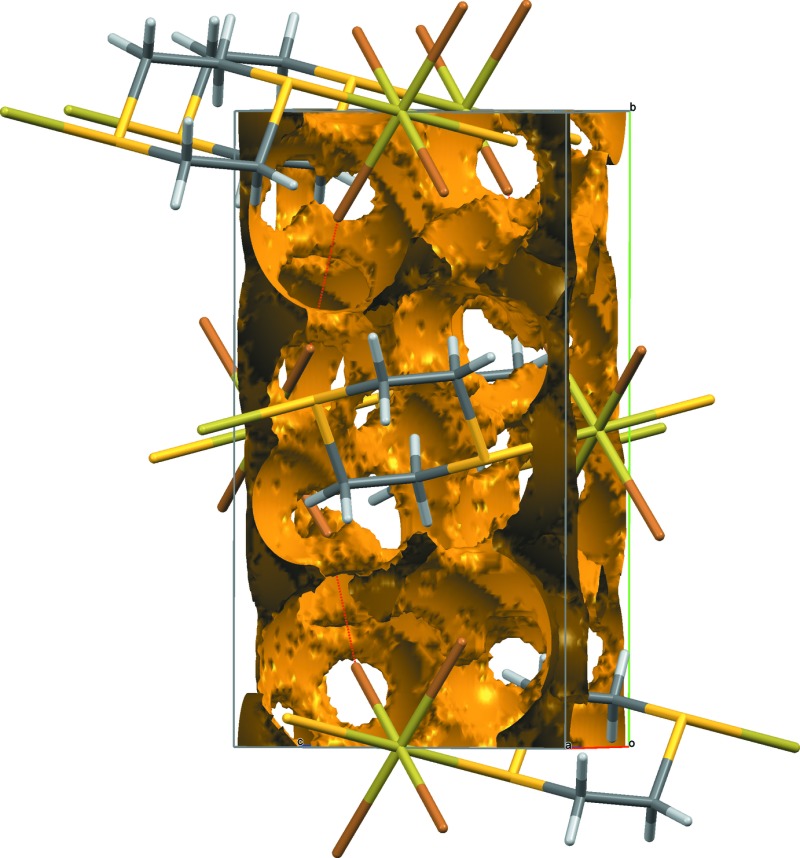
Three-dimensional representation of the contact surface (probe radius = 0.2 Å, outside color = yellow, inside color = brown) within the unit cell visualizing Br···Br interactions (red) between neighboring chains through holes in the surface.

. DOI: 10.1107/S2056989015023932/nr2064fig5.tif
Three-dimensional representation of the contact surface (probe radius = 0.2 Å, outside color = yellow, inside color = brown) within the unit cell visualizing Br⋯Br inter­actions (red) between neighboring chains through holes in the surface.

CCDC reference: 1442283

Additional supporting information:  crystallographic information; 3D view; checkCIF report

## Figures and Tables

**Table 1:** Selected contacts (Å)

Br1⋯H11^i^	2.965
Br1⋯H21^ii^	2.993
Br2⋯H22^iii^	2.944
Br1⋯H12^iv^	3.078
Br1⋯H11^v^	3.079
Br1⋯Br2^vi^	3.5724 (4)

Symmetry codes: (i) 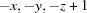; (ii) 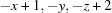; (iii) 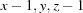; (iv) 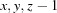; (v) 
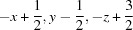
; (vi) 
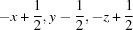
.

## supplementary crystallographic information

### S1. Synthesis and crystallization

A mixture of 0.55 g (2 mmol) SnBr_2_ and 0.24 g (2 mmol) 1,4-di­thiane was heated in a closed ampule to 130 °C for 6 h. No special care was taken to exclude oxygen or humidity. After cooling, the ampule was opened and its solid content inspected by optical microscopy. Only one fragment, a yellow needle-like crystal of the title compound proved to be suitable for single-crystal X-ray diffraction. The presence of tin(IV) in the title compound instead of tin(II) demonstrates the complexity of reactions that must have taken place. Sensitivity of tin(II) compounds towards oxidation by air, however, is not unusual and well documented in literature (*e.g.* Deacon *et al*, 1997).

### S1.1. Refinement

All hydrogen atoms could be localized in difference Fourier syntheses but were refined in geometric positions riding on the carbon atoms with C—H distances of 0.99 Å (–CH_2_–) and one common, free refined isotropic displacement factor.

### S2. Results and discussion

Only some few coordination compounds of tin(IV) bromide with Lewis-bases containing two or more S-atoms as Lewis-base centers have been structurally characterized. The main structural features are one-dimensional chain structures in case of macrocyclic thio­ether complexes (Levason *et al.*, 2003) with the Lewis-base molecules in a *cis*-, and *trans*-position, respectiveley, and the formation of monomeric complexes as a result of chelatization in case of open chain di­thio­ether molecules (Dann *et al.*, 1996). In all cases, the tin atoms are o­cta­hedrally coordinated with similar Sn—Br and Sn—S bond lengths.

### Crystal data

**Table:**

[SnBr_4_(C_4_H_8_S_2_)]	*F*(000) = 508
*M**_r_* = 558.55	*D*_x_ = 3.160 Mg m^−^^3^
Monoclinic, *P*2_1_/*n*	Mo *K*α radiation, λ = 0.71073 Å
*a* = 7.1033 (4) Å	Cell parameters from 9935 reflections
*b* = 12.0526 (8) Å	θ = 3.4–28.7°
*c* = 7.4032 (5) Å	µ = 16.09 mm^−^^1^
β = 112.144 (2)°	*T* = 100 K
*V* = 587.06 (7) Å^3^	Needle, yellow
*Z* = 2	0.16 × 0.06 × 0.06 mm

### Data collection

**Table:**

Bruker APEXII CCD diffractometer	1339 reflections with *I* > 2σ(*I*)
φ and ω scans	*R*_int_ = 0.066
Absorption correction: multi-scan (*SADABS*; Bruker, 2009)	θ_max_ = 28.0°, θ_min_ = 3.4°
*T*_min_ = 0.182, *T*_max_ = 0.450	*h* = −9→9
22217 measured reflections	*k* = −15→15
1426 independent reflections	*l* = −9→9

### Refinement

**Table:**

Refinement on *F*^2^	Secondary atom site location: difference Fourier map
Least-squares matrix: full	Hydrogen site location: inferred from neighbouring sites
*R*[*F*^2^ > 2σ(*F*^2^)] = 0.017	H-atom parameters constrained
*wR*(*F*^2^) = 0.036	*w* = 1/[σ^2^(*F*_o_^2^) + (0.0043*P*)^2^ + 0.5753*P*] where *P* = (*F*_o_^2^ + 2*F*_c_^2^)/3
*S* = 1.14	(Δ/σ)_max_ < 0.001
1426 reflections	Δρ_max_ = 0.72 e Å^−^^3^
54 parameters	Δρ_min_ = −0.46 e Å^−^^3^
0 restraints	Extinction correction: *SHELXL2014*/7 (Sheldrick 2015), Fc^*^=kFc[1+0.001xFc^2^λ^3^/sin(2θ)]^-1/4^
Primary atom site location: structure-invariant direct methods	Extinction coefficient: 0.0042 (3)

### Special details

**Table:**

Geometry. All e.s.d.'s (except the e.s.d. in the dihedral angle between two l.s. planes) are estimated using the full covariance matrix. The cell e.s.d.'s are taken into account individually in the estimation of e.s.d.'s in distances, angles and torsion angles; correlations between e.s.d.'s in cell parameters are only used when they are defined by crystal symmetry. An approximate (isotropic) treatment of cell e.s.d.'s is used for estimating e.s.d.'s involving l.s. planes.

### Fractional atomic coordinates and isotropic or equivalent isotropic displacement parameters (Å^2^)

**Table:**

	*x*	*y*	*z*	*U*_iso_*/*U*_eq_	
Sn1	0.0000	0.0000	0.5000	0.00740 (7)	
Br1	0.17963 (4)	−0.12497 (2)	0.33464 (3)	0.01109 (8)	
Br2	0.04504 (4)	0.16589 (2)	0.30464 (4)	0.01267 (8)	
S1	0.36282 (9)	0.04681 (5)	0.76832 (9)	0.01033 (13)	
C1	0.3132 (4)	0.0756 (2)	0.9867 (3)	0.0119 (5)	
H11	0.2349	0.1455	0.9686	0.015 (4)*	
H12	0.2295	0.0151	1.0078	0.015 (4)*	
C2	0.5088 (4)	0.0860 (2)	1.1657 (4)	0.0129 (5)	
H21	0.4771	0.1146	1.2764	0.015 (4)*	
H22	0.6002	0.1399	1.1388	0.015 (4)*	

### Atomic displacement parameters (Å^2^)

**Table:**

	*U*^11^	*U*^22^	*U*^33^	*U*^12^	*U*^13^	*U*^23^
Sn1	0.00749 (13)	0.00663 (12)	0.00903 (13)	0.00013 (8)	0.00420 (9)	0.00008 (8)
Br1	0.01171 (14)	0.01121 (13)	0.01229 (14)	0.00266 (9)	0.00672 (10)	−0.00103 (9)
Br2	0.01522 (15)	0.00948 (13)	0.01585 (14)	0.00006 (9)	0.00873 (11)	0.00347 (9)
S1	0.0096 (3)	0.0106 (3)	0.0110 (3)	0.0001 (2)	0.0041 (2)	0.0002 (2)
C1	0.0122 (12)	0.0131 (12)	0.0102 (12)	0.0027 (10)	0.0041 (10)	−0.0012 (10)
C2	0.0121 (12)	0.0117 (12)	0.0124 (12)	0.0029 (10)	0.0017 (10)	−0.0038 (10)

### Geometric parameters (Å, º)

**Table:**

Sn1—Br2^i^	2.5574 (3)	Br1—H11^v^	3.0788
Sn1—Br2	2.5574 (3)	Br1—Br2^vi^	3.5724 (4)
Sn1—Br1	2.5638 (2)	S1—C1	1.813 (2)
Sn1—Br1^i^	2.5638 (3)	S1—C2^ii^	1.816 (3)
Sn1—S1^i^	2.6546 (6)	C1—C2	1.521 (3)
Sn1—S1	2.6546 (6)	C1—H11	0.9900
Br1—H11^i^	2.9646	C1—H12	0.9900
Br1—H21^ii^	2.9932	C2—S1^ii^	1.816 (3)
Br2—H22^iii^	2.9438	C2—H21	0.9900
Br1—H12^iv^	3.0783	C2—H22	0.9900
			
Br2^i^—Sn1—Br2	180.0	H21^ii^—Br1—H11^v^	68.8
Br2^i^—Sn1—Br1	90.092 (9)	H12^iv^—Br1—H11^v^	143.5
Br2—Sn1—Br1	89.908 (9)	Sn1—Br1—Br2^vi^	167.821 (10)
Br2^i^—Sn1—Br1^i^	89.908 (9)	H11^i^—Br1—Br2^vi^	102.6
Br2—Sn1—Br1^i^	90.092 (9)	H21^ii^—Br1—Br2^vi^	88.3
Br1—Sn1—Br1^i^	180.0	H12^iv^—Br1—Br2^vi^	85.5
Br2^i^—Sn1—S1^i^	87.931 (15)	H11^v^—Br1—Br2^vi^	58.0
Br2—Sn1—S1^i^	92.069 (15)	Sn1—Br2—H22^iii^	79.0
Br1—Sn1—S1^i^	92.027 (15)	C1—S1—C2^ii^	100.11 (12)
Br1^i^—Sn1—S1^i^	87.973 (15)	C1—S1—Sn1	104.35 (8)
Br2^i^—Sn1—S1	92.069 (15)	C2^ii^—S1—Sn1	105.15 (8)
Br2—Sn1—S1	87.931 (15)	C2—C1—S1	111.83 (17)
Br1—Sn1—S1	87.973 (15)	C2—C1—H11	109.2
Br1^i^—Sn1—S1	92.027 (15)	S1—C1—H11	109.2
S1^i^—Sn1—S1	180.00 (3)	C2—C1—H12	109.2
Sn1—Br1—H11^i^	83.1	S1—C1—H12	109.2
Sn1—Br1—H21^ii^	83.6	H11—C1—H12	107.9
H11^i^—Br1—H21^ii^	161.5	C1—C2—S1^ii^	111.25 (17)
Sn1—Br1—H12^iv^	106.2	C1—C2—H21	109.4
H11^i^—Br1—H12^iv^	80.0	S1^ii^—C2—H21	109.4
H21^ii^—Br1—H12^iv^	116.1	C1—C2—H22	109.4
Sn1—Br1—H11^v^	110.3	S1^ii^—C2—H22	109.4
H11^i^—Br1—H11^v^	104.2	H21—C2—H22	108.0
			
Br2^i^—Sn1—S1—C1	−60.87 (9)	Br1—Sn1—S1—C2^ii^	−46.00 (9)
Br2—Sn1—S1—C1	119.13 (9)	Br1^i^—Sn1—S1—C2^ii^	134.00 (9)
Br1—Sn1—S1—C1	−150.89 (9)	Sn1—S1—C1—C2	170.73 (16)
Br1^i^—Sn1—S1—C1	29.11 (9)	S1—C1—C2—S1^ii^	−69.0 (2)
Br2^i^—Sn1—S1—C2^ii^	44.02 (9)	C1—C2—S1^ii^—C1^ii^	61.7 (2)
Br2—Sn1—S1—C2^ii^	−135.98 (9)	C2^ii^—S1—C1—C2	62.1 (2)

Symmetry codes: (i) −*x*, −*y*, −*z*+1; (ii) −*x*+1, −*y*, −*z*+2; (iii) *x*−1, *y*, *z*−1; (iv) *x*, *y*, *z*−1; (v) −*x*+1/2, *y*−1/2, −*z*+3/2; (vi) −*x*+1/2, *y*−1/2, −*z*+1/2.
