# Supplementary figures and images for: Crystal structure of a one-dimensional coordination polymer of tin(IV) bromide with 1,4-di­thiane
Source: Acta Crystallogr E Crystallogr Commun. 2015 Dec 16;71(Pt 12):m267–8. doi: 10.1107/S2056989015023932 (PMC4719867; doi:10.1107/S2056989015023932)

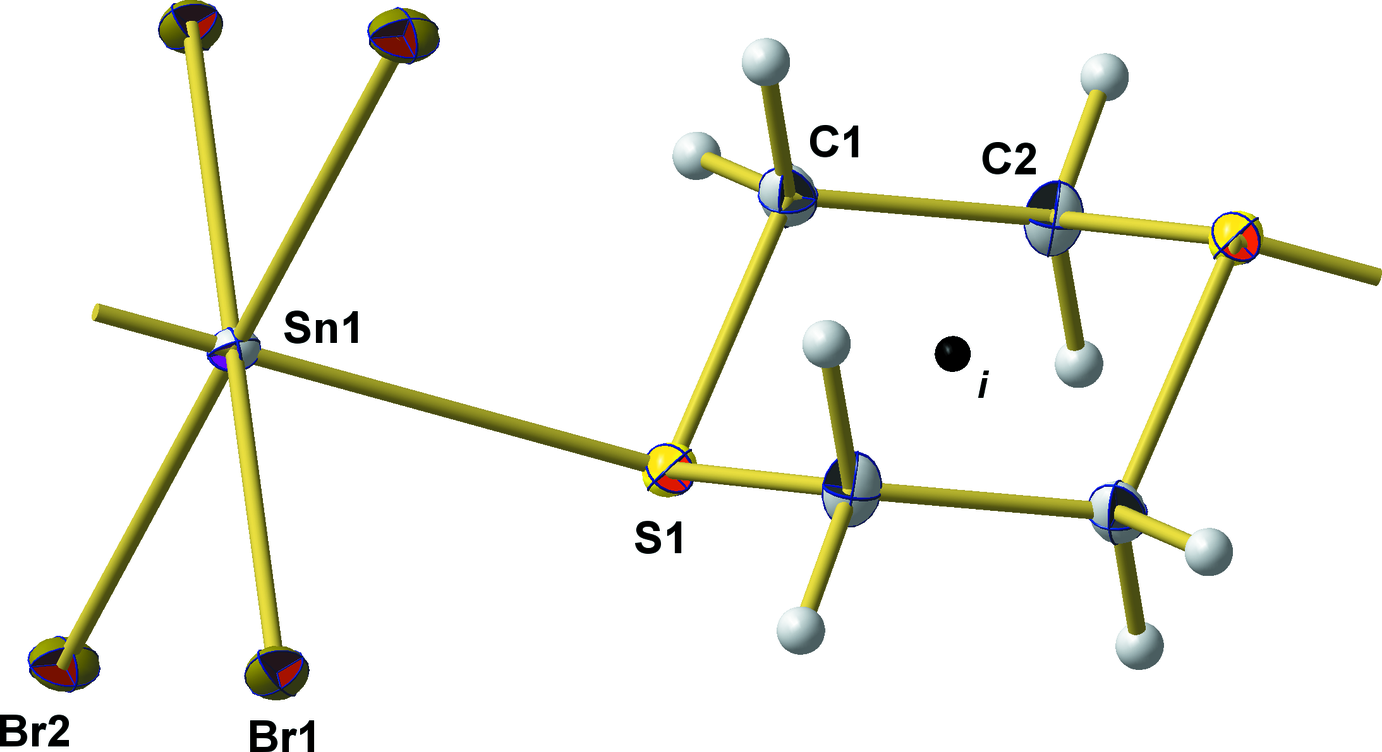

Supplement: Supplementary file 3 [file e-71-0m267-fig1.tif]

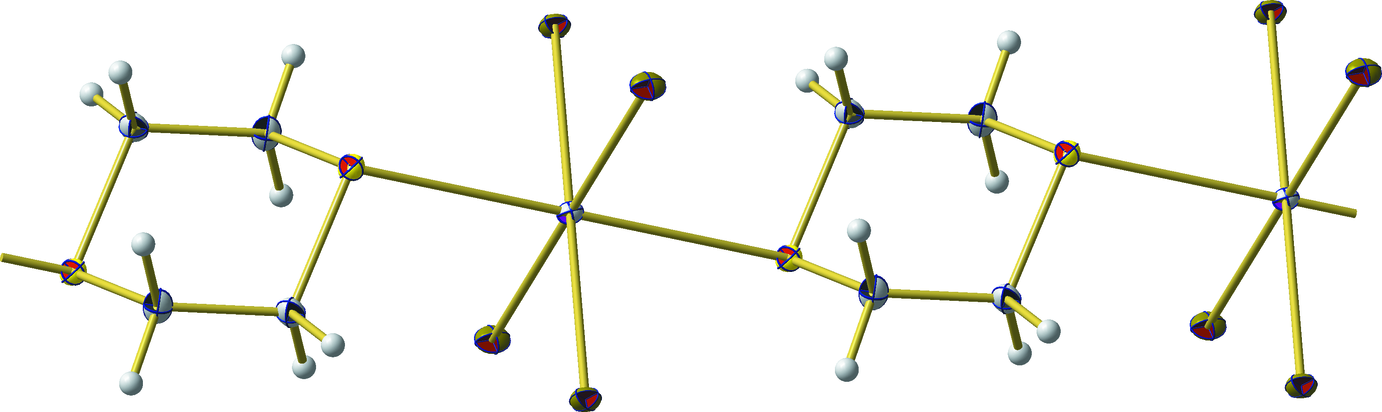

Supplement: Supplementary file 4 [file e-71-0m267-fig2.tif]

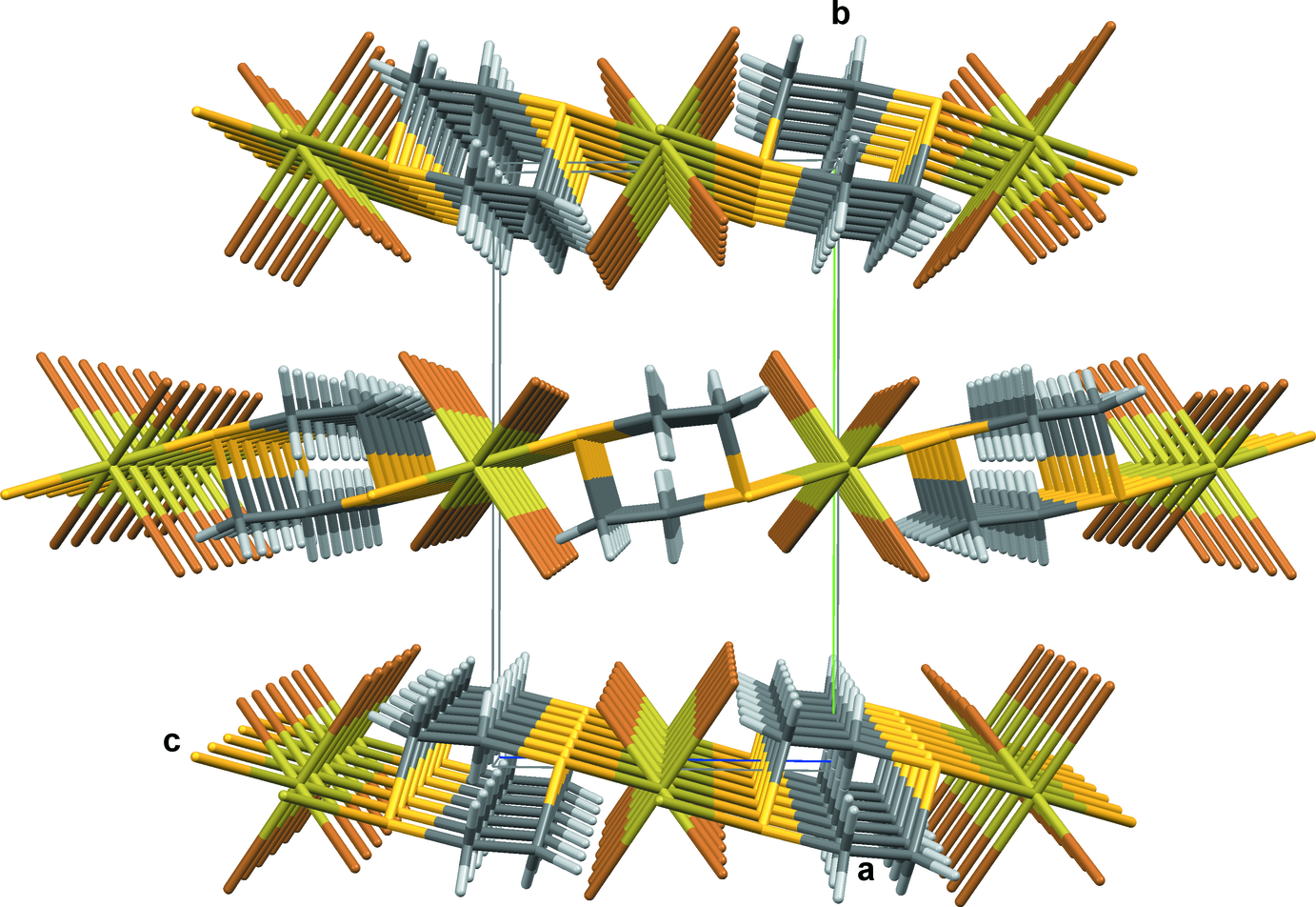

Supplement: Supplementary file 5 [file e-71-0m267-fig3.tif]

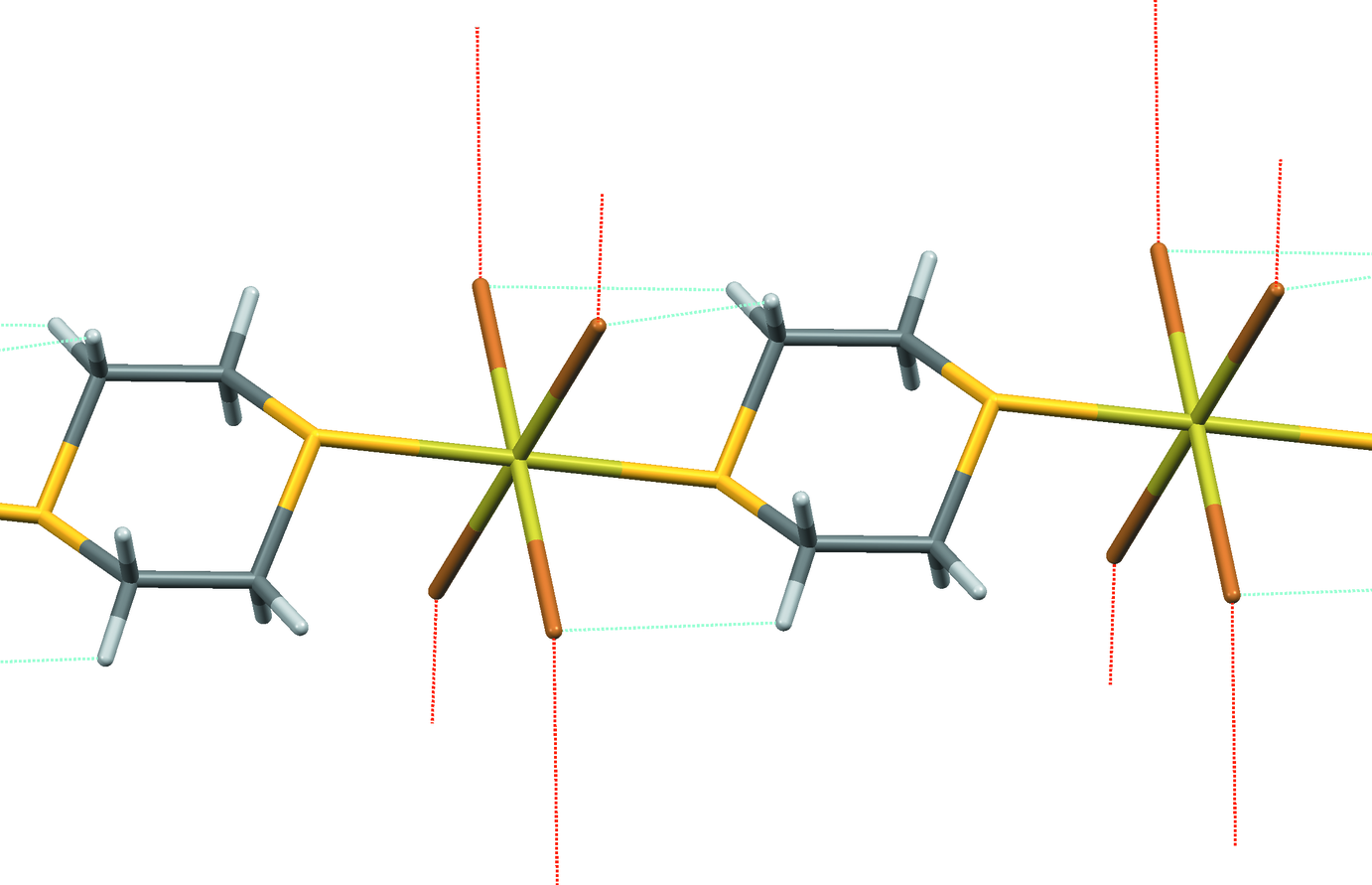

Supplement: Supplementary file 6 [file e-71-0m267-fig4.tif]

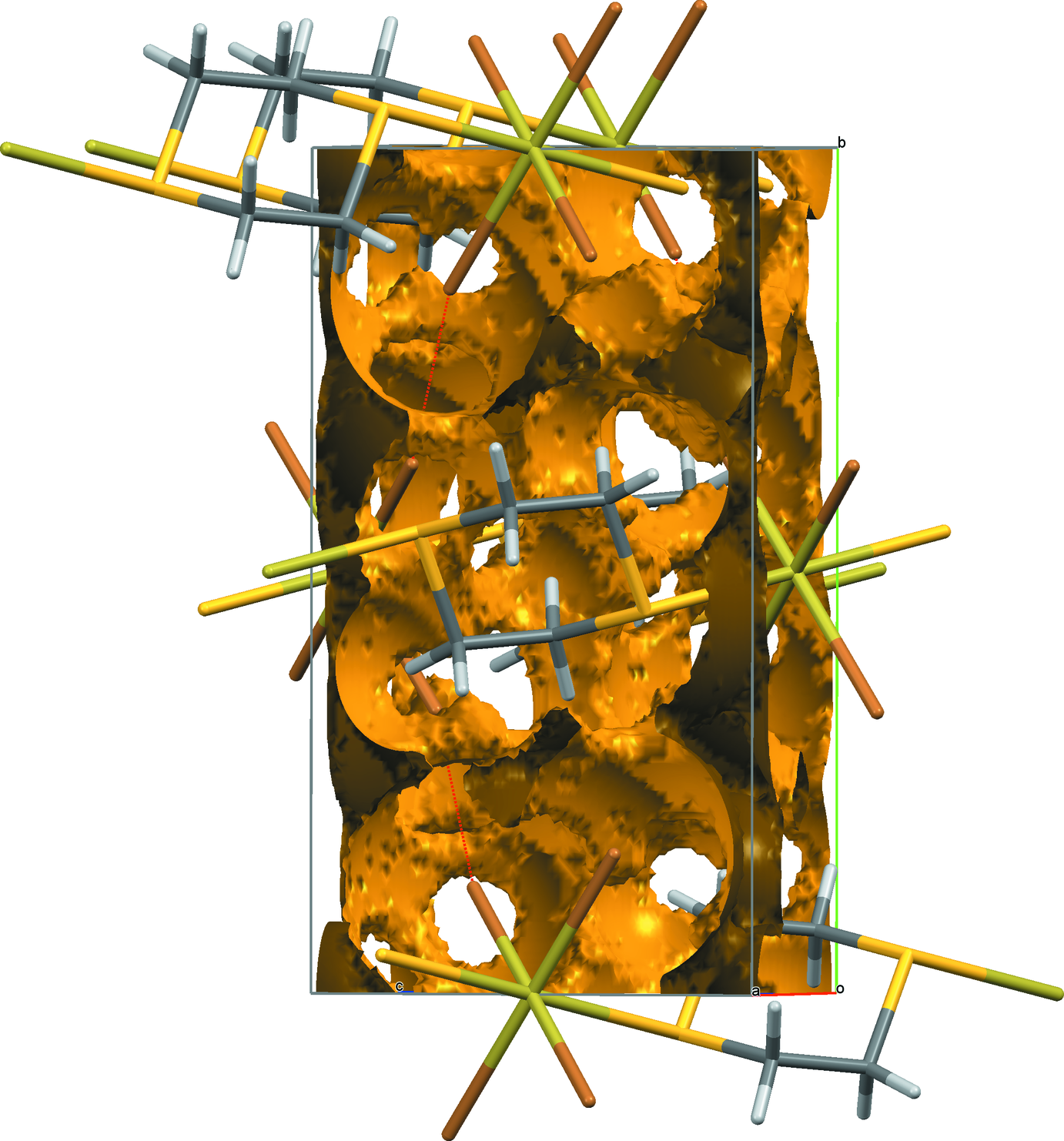

Supplement: Supplementary file 7 [file e-71-0m267-fig5.tif]
